# Survival status of hiv positive adults on antiretroviral treatment in Debre Markos Referral Hospital, Northwest Ethiopia: retrospective cohort study

**DOI:** 10.11604/pamj.2014.17.88.3262

**Published:** 2014-02-03

**Authors:** Nurilign Abebe, Kassahun Alemu, Tadese Asfaw, Amanuel Alemu Abajobir

**Affiliations:** 1 Health Sciences College, Debre Markos University, Debre Markos, Ethiopia; 2 College medicine and Health Sciences, University of Gondar, Gondar, Ethiopia

**Keywords:** Survival status, HIV, antiretroviral treatment, Ethiopia

## Introduction

Globally an estimated 34 million people were living with HIV at the end of 2010 [1]. The introduction of antiretroviral (ARV) in 1996 was a turning point for hundreds of thousands of people with access to sophisticated healthcare. United Nation for International Development (UNAID) and World Health Organization (WHO) estimates that since the availability of effective treatment some 2.9 million lives have been saved [2–4]. About 68% of all people living with HIV resided in sub-Saharan Africa, a region with only 12% of the global population in 2010 [5]. Ethiopia is among the seriously affected countries in sub Saharan Africa with more than 1.3 million people living with HIV and an estimated 277,800 people requiring treatment [1]. The overall adult HIV prevalence was 1.4% and 1.5% in 2005 and 2011 [6–8]. The government of Ethiopia launched its fee-based ART initiative in 2003 and free-ART initiative in 2005. Persons ever started ART were 150,136, 208,784 and 268,934 respectively in 2008, 2009 and 2010; persons who were on ART on the same years were 109,930, 152,472 and 207,733 respectively [6–9].

The situation of HIV/AIDS in the study region is one of the worst in the country with persistently high prevalence. The regions prevalence was estimated at 2.9% in 2010. Currently, there are an estimated 379,096 people living with HIV/AIDS in the region [10]. Information on survival from Africa is limited and comes from studies of short duration with relatively high loss to follow-up [11].

The effectiveness of HAART could vary from region to region because of the difference in background disease burden (such as tuberculosis or intestinal parasites), viral subtypes and possible genetic differences in drug metabolism [12].

The contribution of ART is paramount in improving patient survival and has to be empowered further by studying its outcome. Therefore, study of the outcome of ART so as to provide necessary information to healthcare providers, policy makers and program funders to determine resource allocation and optimize care and treatment strategies for HIV in Ethiopia is timely issue.

Thus, this study was aimed to determine the survival status and its predictors among HIV positive adults on ART in Debre Markos Referral Hospital, Northwest Ethiopia, and 2005-2013.

## Methods

### Study design, area and period

A facility-based retrospective cohort study design was conducted in Debre Markos Referral Hospital from 30^th^ September to 30^th^ February 2013.

### Study population

All HIV positive adults’ record in Debre Markos Referral Hospital who were on antiretroviral therapy enrolled to treatment from 30^th^ September 2005 to 30^th^February 2013 were the source population and selected HIV positive adults’ record on care and support follow up who had started ART at the hospital within the same period were included in the study.

### Eligibility criteria

**Figure 1:**
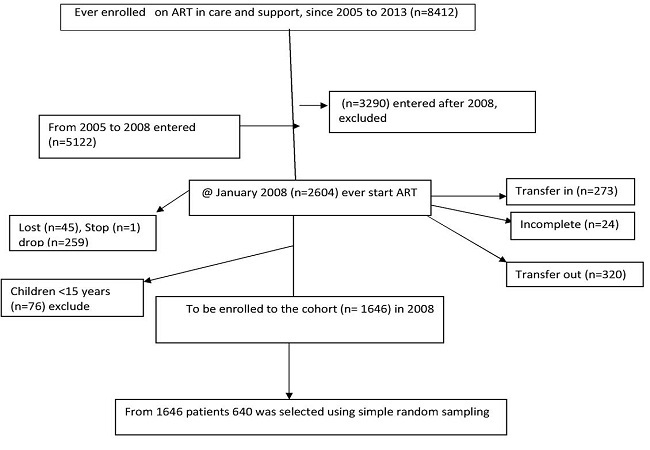
Schematic representation of sampling technique of HIV patients on ART, in Debre Markos referral hospital, 2005-2013

All adult (> = 15 years old) HIV positive individuals on care and support follow up who had started ART and had at least one visit in Debre Markos referral hospital were included. Adults with incomplete registration cards during the review, who started ART from other healthcare institutions, drop outs, lost and transfer, were excluded from the study (Figure 1).

### Sample size determination

As the investigation was cohort study, the sample size required for achieving statistically significant results was determined using two population proportion formula. Therefore, sample size was calculated by taking into account the major exposure variables and using open epi version 3.04.04 statistical package [13]. Among exposure variables, WHO staging is chosen as main exposure variable of non-accidental mortality during the 7 years of follow up since it was considered to give the optimal sample size and most significant result. In this regard, a 5% level of significance (two-sided), a power of 80% and a ratio of unexposed to exposed of 1:1, estimated proportion of mortality in Ethiopia was taken 4.1% for non-exposed group (WHO stage I and II) and 10.1% for exposed group (WHO stage III and IV) [14]. (Table 1). However, in practice getting 320 patients in their WHO stage I and II was difficult and the rest were from stage III and IV. Thus, the total sample size was 640 [12, 15].

**Table 1:** Sample size calculation of retrospective cohort study HIV patients on ART in Debre Markos Referral Hospital, Northwest Ethiopia, 2005- 2013

Assumptions		Major exposure variables	Sample size by Fleiss with CC Formula	Total number of sample size
**Two-sided significance level:**	0.05	WHO clinical HIV staging grouped (I, II non exposed; III, IV exposed)	Number of exposed = 320	640
**Power:**	80			
**Ratio of sample size:**	1:1		Number of non- exposed = 320	
**Percent of Unexposed with Outcome**	4.1			
**Percent of Exposed with Outcome**	10.1			
**Odds Ratio**	0.38			
**Relative risk**	0.41			

### Sampling technique

**Figure 2:**
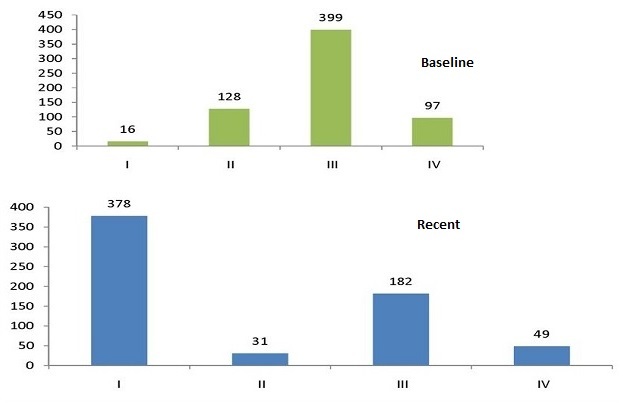
Baseline and recent WHO clinical stages of patients on ART in Debre Markos Referral Hospital, 2005-2013

Simple random sampling technique was used to recruit predetermined sample size from the clinic computerized register. First registration number was identified and computer generated number was used to select study subjects among the eligible cards (Figure 2).

### Variables of the study

**Dependent variable:** Survival status

### Independent variables

**Demographic factors:** age, sex, residence, religion, marital status, ethnicity, educational status

**Clinical conditions:** WHO staging, base line CD^+^_4_ counts, Hgb status, opportunistic infections, concomitant illness, diagnosis/functional status, cotrmoxazole prophylaxis, ART

**Regimen:** Types of first line, second line, change in regimen

**Follow up outcomes:** Lost to follow up, transfer out, adherence

### Operational definition

**Incomplete card:** when one of the independent variable is not registered namely, CD4+ cells, Hgb, WHO stage, functional status and TB status

**Lost to follow up:** not seen since >/= 1 month < 3 months [12, 15].

**Drop:** lost to follow up for >3 months

**Transfer out:** a patient is referred to another health facility for care evidenced by his/her document.

**Functional Status:** Working: able to perform usual work in or out of the house; Ambulatory: able to perform activities of daily living; Bedridden: not able to perform activities of daily living [12, 15].

**Survival:** when the patient is known to be alive as evidenced by hi/her clinical follow up till the end of the study period.

**Survival time:** the period that a patient stays in life after starting ART.

**Adherence:** Good: if the percentage of missed dose is between <95% (<2 doses of 30 doses or < 3 dose of 60 dose) as documented by ART physician; Fair: if the percentage of missed dose is between 85-94% (3-5 doses of 30 doses or 3-9 dose of 60 dose) as documented by ART physician;Poor: if the percentage of missed dose is between < 85% (>6 doses of 30 doses or > 9 dose of 60 dose) as documented by ART physician;

**Side effect:** as recorded by ART physician/nurse on the patient card.

### Data collection procedure

Data collecting checklist was prepared based on routine data registration protocol using the standardized ART entry and follow up form employed by the ART clinic. The data collecting checklist was used by data collectors for recording information from patients’ cards

### Data quality control

To ensure quality, data were collected by ART staff nurses working in the hospital after one day training on the techniques of data collection. The completeness of data was checked by two trained supervisors so as to provide feedback in registration process and to correct when necessary. Furthermore, every night data collectors, supervisors and principal investigators used to discuss about documenting the findings and exchange of the information. Moreover, pre-test was done on registrations that were not included in the final study.

### Data processing and analysis

The data were entered in Epi data version 3.1 computer program. Prior to the analysis, the whole data were cleaned and 20% of the data was double-entered. The completeness of the data was checked. Errors related to inconsistency were verified using cross tabulation and other data exploration methods. The data was exported to Statistical Package for Social Sciences (SPSS) version 16.0. Then recoded, categorized and sorted to facilitate analysis. The data was analyzed using SPSS version 16.0. Kaplan-Meier model was used to estimate survival probability after ART initiation and log rank tests was used to compare survival curves. Those variables showed statistical significance in univariate analysis with p- value of <0.25 [16] were selected for multivariable analysis and declared as statistically significant when p-value <0.05.

### Ethical consideration

Ethical clearance was obtained from the Ethical Review Board of Debre Markos University, College of Health Sciences, and department of Public Health. Permission was obtained from Debre Markos Referral Hospital and ART clinic. Verbal informed consent was obtained from responsible bodies of the hospital and ART clinic prior to the review. Confidentiality and privacy of the information were assured and maintained.

## Results

Between September 19, 2005 to January 30, 2008, 5122 HIV patients ever enrolled to Debre Markos Referral Hospital from which 2604 were on ART.

### Basic characteristics of the cohort

Cards of six hundred forty (379 alive and 261 death) adult HIV infected individuals were included in the present study. Among the cohort, 53.1% was female and the mean age was 35.40 years (SD±9.62). Five hundred ninety five (93%) of the study participants were followers of orthodox and 260 40.8% were married. Two hundred thirty four (36.6%) of the study participants had primary education. More than half, 353 (55.2%), of the study subjects were unemployed. The median weight at base line was 50 kg (IQR= 44-55 kg). Base line mean hemoglobin was 12.23 (IQR= 10.6-14). The base line median CD4 count was 115 cells/µl (IQR= 63.25-164.75). About 399 (62.3%) were at WHO clinical stage III during the initiation of ART. Moreover, 415 (64.8%) of the participants were in working functional status and 196 (30.6%) were ambulatory in the time of ART initiation (Table 2, Table 3). Baseline opportunistic infections were recorded in the patient registration; 92 (14.38%), 6 (0.94%), 234 (36.562%), 203 (31.79%) and 381 (59.53%) had esophageal candidacies, Kaposi's sarcoma, oral recurrent candidacies, unexplained persistent diarrhea and fever respectively. Twenty four patients (3.75%) had presumed diagnosis of PCP. Patients with unexplained weight loss >10% were 103 (16.09%) and patients with recurrent severe bacterial pneumonia, active TB, herpes simplex, recurrent upper respiratory tract infections and herpes zoster were 197 (30.78%), 89 (13.91%), 94 (1.68%), 192 (30%) and 101 (15.75%) respectively.

**Table 2:** Socio-demographic characteristics of patients initiated ART at Debre Markos Referral Hospital during 2005-2013 (N = 640).

Variables	Alive (n = 379)	Death (n =261)
**Sex**		
Male	176 (46.4%)	124 (47.5%)
Female	203 (53.6%)	137 (52.5%)
**Age in years**		
15-29	107 (16.7%)	82 (12.8%)
30-44	214 (33.4%)	118 (18.4%)
45-59	52 (8.1%)	55 (8.6%)
>= 60	6 (0.9%)	6 (0.9)
**Residence**		
Urban	275 (43.0%)	181 (28.3%)
Rural	104 (16.2%)	80 (12.5%)
**Ethnicity**		
Amhara	369 (57.7%)	258 (40.3%)
Oromo	5 (0.8)	1 (0.2%)
Tigray	4 (0.6%)	2 (0.3%)
Others	1 (0.2%)	0 (0%)
**Marital status**		
Married	168 (26.2%)	93 (14.5%)
Widowed	77 (12%)	47 (7.3%)
Never married	41 (6.4%)	32 (5%)
Divorced	77 (12%)	71 (11.1%)
Separated	16 (2.5%)	18 (2.8%)
**Educational status**		
Not educated	89 (13.9%)	78 (12.2%)
Primary	149 (23.3%)	85 (13.3%)
Secondary	94 (14.7%)	66 (10.3%)
Tertiary	47 (7.3%)	32 (5%)
**Occupation status**		
Merchant	87 (13.6%)	38 (5.9%)
NGO employee	10 (1.6%)	7 (1.1%)
Gove's employee	82 (12.8%)	63 (9.8%)
Day laborer	69 (1o.8%)	48 (7.5%)
“Jobless”	27 (4.2%)	13 (2%)
Farmer	53 (8.3%)	47 (7.3%)
Others	51 (8.0%)	45 (7%)

**Table 3:** Base line clinical and laboratory information of 640 patients Initiated ART at Debre Markos Referral Hospital during 2005 to 2013

Variables	Alive (n = 379)	Death (n = 261)
**Base line prophylaxis**		
Not given	35 (9.2%)	38 (14.6%)
Cotrmoxazole	338 (89.2%)	212 (81.2%)
INH	4 (1.1%)	7 (2.7%)
Fluconazole	2 (0.5%)	4 (1.5%)
**Base line weight (kg)**		
<60	333 (87.9%)	237 (90.8%)
≥60	46 (12.1%)	24 (9.2%)
**Base line CD4+ (cells/µl)**		
<50	55 (14.5%)	66 (25.4%)
50-99	74 (19.5%)	64 (24.6%)
100-200	205 (54.1%)	108 (41.5%)
201-350	44 (11.6%)	22 (8.5%)
>350	1 (0.3%)	0 (0%)
**Base line Hemoglobin (g/dl)**		
<10	34 (11.3%)	69 (33.2%)
≥10	268 (88.7%)	139 (66.8%)
Not recorded	77 (12%)	53 (8.28%)
**Functional Status**		
Working	299 (78.9%)	125 (47.9%)
Ambulatory	116 (44.4%)	9 (2.40%)
Bedridden	71 (18.7%)	20 (7.7%)
**WHO staging**		
Stage I & II	109 (17.03%)	35 (5.47%)
Stage III & IV	270 (42.2%)	226 (35.31%)
**ART eligibility criteria**		
WHO staging	40 (10.6%)	17 (6.5%)
Immunologic	108 (28.5%)	48 (18.4%)
Both	231 (60.9%)	196 (75.1%)
**Side effect**		
Yes	212 (55.9%)	25 (9.6%)
No	167 (44.1%)	236 (90.4%)
**ART adherence**		
Good	369 (97.4%)	215 (82.4%)
Fair	4 (1.1%)	17 (6.5%)
Poor	6 (1.6%)	29 (11.1%)

### Survival analysis

The cohort's median survival duration was 58 months ranging from 1 month to 105 months. Kaplan-Meier survival estimation showed that overall estimated survival duration after ART initiation was 65.22 (95%CI: 61.409-69.043) months.

The study participants contributed 2430.17 person-years of observation that resulted in incidence density of 10.74% death. Two hundred sixty one (40.78%) patients died in the study period, from which 205 (32.01%) of death occurred in the first 12 months; however, 379 (59.22%) survived till the end of the study.

Kaplan-Meier analysis of survival status showed that male sex show better survival than female; i.e., estimated survival was 64.67 months (95% CI: 59.073-70.270) vs. (61.65 months, (95%CI = 56.863-66.437). Advanced age >60 years indicated lower survival time (47.250 months, 95%CI: 23.390-71.110). From baseline clinical characteristics of patients, those with cotrmoxazole prophylaxis survived better, 67.273 months (95%CI: 63.107-71.439) than those without prophylaxis (47.769 estimated months, 95%CI: 37.749-57.789), patients with lower Hgb <10g/dl, poor adherence showed lesser survival than those with higher >10g/dl Hgb and good drug adherence respectively (Table 4, Table 5).

**Table 4:** Kaplan-Meier Analyses of Survival Status for Patients on Antiretroviral Treatment by Socio-demographic Characteristics of HIV patients, in Debre Markos Referral Hospital 20005-2013.

Variables	Estimated survival in mon	Standard error	Confidence interval (CI 95%)
**Sex**			
Male	64.672	2.857	59.073-70.270
Female	61.65	2.44	56.863-66.437
**Age in years**			
15-29	58.947	3.294	52.490-65.404
30-44	69.866	2.705	64.565-75.168
45-59	51.981	4.092	43.961-60.001
>= 60	47.250	12.174	23.390- 71.110
**Religion**			
Orthodox	65.728	2.011	61.788-69.669
Protestant	77.125	10.173	57.187-97.063
Muslim	43.721	7.019	29.964-57.479
Other	30.000	18.385	0.001-66.034
**Residence**			
Urban	63.287	3.577	56.277-70.297
Rural	62.609	2.076	58.541-66.677
**Marital status**			
Married	69.684	3.035	63.736-75.633
Widowed	61.118	3.947	53.382-68.854
Never married	56.089	4.715	46.847-65.331
Divorced	51.930	3.429	45.210-58.650
Separated	51.397	6.982	37.713-65.082
**Educational status**			
Not educated	52.908	3.302	46.436-59.381
Primary	62.897	2.740	57.526-68.269
Secondary	64.865	3.849	57.320-72.409
Tertiary	59.169	4.367	50.610-67.728
**Occupation status**			
Merchant	68.180	3.548	61.226-75.135
NGO employee	59.875	9.588	41.081-78.668
Gove's employee	63.245	4.045	55.317-71.172
Day laborer	60.964	3.886	53.348-68.581
“Jobless”	65.608	6.166	53.522-77.695
Farmer	50.125	4.188	41.917-58.333
Others	51.189	4.313	42.736-59.642

**Table 5:** Bivariate and multivariate Cox-regression analysis of clinical characteristics of the cohort studied (n = 640 patients) in Debre Markos Referral Hospital, during September 2005 to 2013.

Variables	Frequency		CHR ( 95% CI)	AHR ( 95% CI)	P- value
**Base line prophylaxis**	**Alive**	**Died**			
Not given	35	38	1	1.758 (.610-5.067)	.212
Cotrmoxazole	338	212	1.235 (0.743-0.945)	.837 (.524-1.339	.296
INH	4	7	0.668 (0.552-2.767)	2.948 (.499-17.428	.458
Fluconazole	2	4	1.247 (0.445-3.496)		.233
**Base line weight (kg)**					
<60	333	237	1.374(0.902-2.092)	1.029 (.576-1.838)	.922
≥60	46	24	1	1	
**Hemoglobin (g/dl)**					
<10	34	69	2.762(2.064-3.697)	1.869 (1.319-2.649)	0.000*
≥10	268	139	1	1	
**Functional Status**					
Working	299	125	1		0.000*
Ambulatory	116	9	3.243 (2.513-4.185)	2.727 (1.905-3.905)	0.000*
Bedridden	71	20	4.181 (2.593-6.740)	2.382 (1.326-4.279)	0.004*
**Base line CD4+ (cells/µl)**					
<50	100	21	1	1	0.66
50-99	130	8	0.751(0.532-1.059)	1.021 (0.635-1.643)	0.931
100-200	301	12	0.513(0.378-0.697)	1.112 (0.713-1.734)	0.641
≥201	60	7	0.478(0.295-0.774)	0.705 (0.342-1.456)	0.345
**WHO staging**					
Stage I &II	109	35	1	1	
Stage III&IV	270	226	2.238 (1.567-3.196)	2.164 (1.100-4.258)	0.025*
**ART eligibility criteria**					
WHO staging	40	17	1	1	.600
Immunologic	108	48	1.046 (0.601-1.818)	1.504 (.677-3.340)	.316
Both	231	196	1.816 (1.106-2.982)	1.286 (.692-2.392)	.426
**Side effect**					
Yes	212	25	1	1	
No	167	236	8.021 (5.310-12.14)	7.816 (4.589-13.310)	0.000*
**ART adherence**					
Good	369	215	1	1	.019*
Fair	4	17	3.495 (2.126-5.743)	2.169 (1.030-4.567)	.042*
Poor	6	29	4.102 (2.767-6.080)	1.887 (1.081-3.295)	.025*

**Figure 3:**
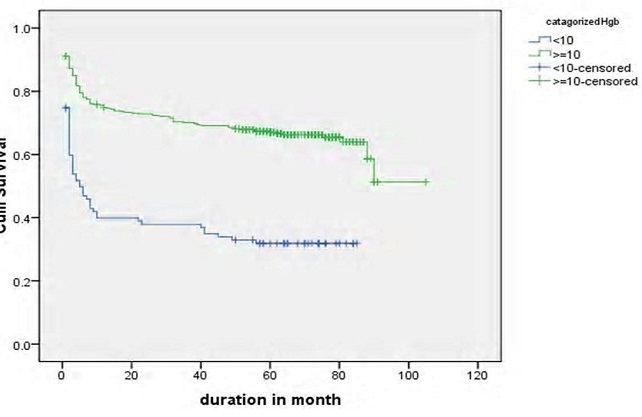
Survival functions of patients on ART by baseline hemoglobin in Debremarkos Referral Hospital, 2005-2013 (Log rank test p < 0.001)

**Figure 4:**
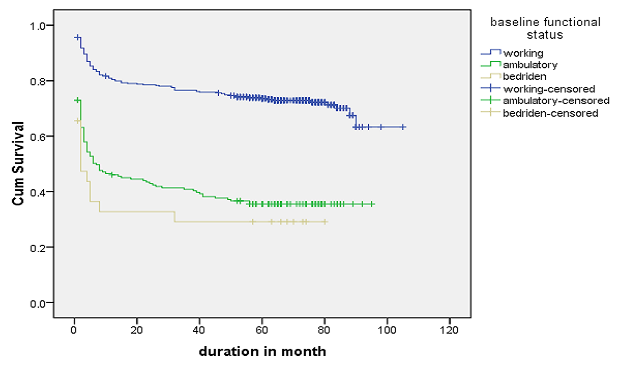
Patterns of survival function of patients on ART treatment in Debre Markos Referral Hospital by base line functional status, 2005-2013 (Log rank test p < 0.001)

**Figure 5:**
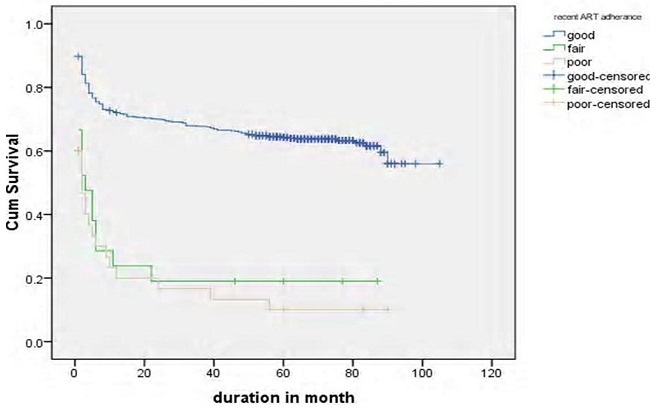
Median survival of patients based on their recent ART adherence (Log rank test p < 0.000)

**Figure 6:**
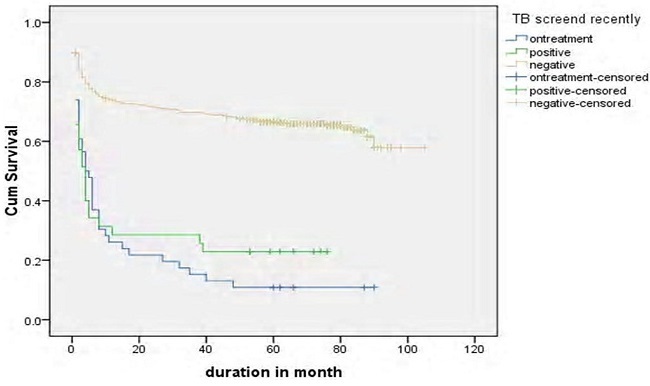
Median survival of patients on ART by TB status in Debre Markos Referral Hospital, 2005-2013 (Log rank test p < 0.000)

**Figure 7:**
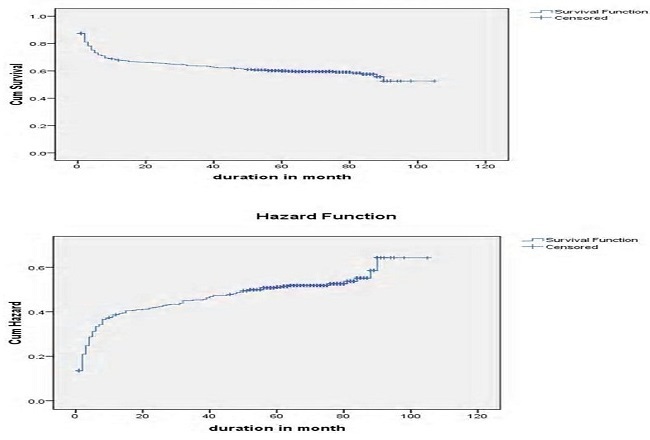
Overall Kaplan-Meier estimation of survival a) and hazard b) functions of patients on ART in Debre Markos Referral Hospital, 2005-2013

Log rank test for different groups of patients showed that the difference in survival and hazard curves. Base line Hgb, baseline functional status, recent adherence of patients to the drug and presence of OIs were shown in Figure 3. Patients based age category, availability of OI prophylaxis, baseline CD4 count and presence of toxoplasmosis, WHO staging on treatment, and TB screened status were some of the variables that showed significance in Log rank test (Figure 4, Figure 5,Figure 6, Figure 7).

In multivariate Cox regression analysis those variables with p-value <0.25 in the bivariate analysis and non-collinear independent variables were included. The variables selected for multivariable analysis were age, occupational status, educational status, religion, marital status, types of OI prophylaxis at baseline, baseline functional status, unexplained chronic diarrhea >1 month, weight loss >10%, baseline Hgb, baseline CD4 count, recent treatment adherence, baseline WHO clinical staging and other opportunistic infections. Among these variables, none of the socio-demographic variables was significant predictor of mortality

## Discussion

In this retrospective cohort study, the survival probability was increased from early phase of ART follow up to the end of the study. Over all estimated survival of patients after ART was 65.22 months.

In this study after initiation of the antiretroviral treatment, HIV-positive patients lived for an average of 65.22 (95% CI = 61.409-69.043) months. This finding was in agreement with studies in other African countries and in Ethiopia [17–19] where average survival of patients after ART was 63.7 month [17] and 77 months [18]. After initiation of the antiretroviral treatment, HIV-positive patients lived for an average of 5.65 years [19]. However, the mean survival of patients after ART initiation was 43 months in SNNPR, Ethiopia [15]. This may be because of the relatively short period of time in the later study (4 year) in which death is very high in early periods of treatment and more than 1000 subjects were in WHO stage IV. This study also shows that relatively short average duration of study subjects (2.03 deaths per 100 person years). Also the above slight difference (65 vs. 77) might be the later study was done in Military Hospital in Addis Ababa, Ethiopia. This is might also be because of better awareness of patients, better clinical care and nutrition, and socio-demographic differences compared with the current study areas.

Patients with side effect survive more than those patients with no recorded side effects. Probably this is due to high mortality rate in early periods of treatment in which side effects are not commonly seen.

Kaplan-Meier analysis of survival status showed that male sex show better survival than female i.e estimated survival was 64.67 months (95% CI= 59.073-70.270) Vs (61.65 months, (95%CI = 56.863-66.437). Advanced age >60 years indicate lower survival time (47.250 months, 95% CI = 23.390-71.110). From baseline clinical characteristics of patients, those with cotrmoxazole prophylaxis survives better 67.273 months (95%CI = 63.107-71.439) than those without prophylaxis (47.769 estimated months, 95% CI = 37.749-57.789) (Table 6). In the present study, patients with hemoglobin <10g/dl at base line was at high risk of death. Study on cause's specific mortality indicators study from LMIC [20]. Study in Tanzania rural hospital by Johannessen A. etal [21]. Studies in Ethiopia [22–25] indicated that patients with anemia were at high risk of death after ART initiation. The possible explanation for this phenomenon could be 234 of patients took ZDV which is responsible factor for persistent anemia as indicated in other study [16]. As a result, patients with lower Hgb should be closely followed and monitor ZDV drug administration.

**Table 6:** Bivariate and multivariate Cox Regression analysis of base line opportunistic infection among ART follower patients (n = 640) in Debre Markos Referral Hospital, during 2005 to 2013.

Opportunistic infections	Frequency		CHR (95% CI)	AHR (95% CI)	P value
**Esophageal candidacies**	**Alive**	**Died**			
Yes	3	31	1	1	.580
No	30	184	0.681 (0.464-1.00)	.894 (.601-1.330)	
**Oral recurrent candidacies**					
Yes	9	73	1	1	.064**
No	24	142	0.484 (0.379-0.618)	1.356 (.983-1.872)	
**Unexplained chronic diarrhea >1 mon**					
Yes	4	68	1.437 (1.077-1.921)	1.535 (1.094-2.155)	.013*
No	29	147	1	1	
**Unexplained presumed wt loss >10%**					
Yes	2	23	1	1	
No	31	193	0.594 (0.442-0.799)	.765 (.527-1.111)	.159
**Severe bacterial infection pneumonia**					
Yes	55	45	1	1	
No	28	170	0.556 (0.324-0.952)	.545 (.244-1.218)	.139
**Recurrent URTI**					
Yes	4	27	1	1	.673
No	30	189	0.821 (0.635-1.061)	.933 (.677-1.286)	
**Herpes zoster**					.194
Yes	61	39	1.015 (0.725-1422)	1.328 (.866-2.037)	
No	318	221	1	1	
**TB screened during ART start**					
**On treatment**	5	41	3.901 (2.773-5.489)	2.331 (.823-6.601)	.276
Positive	8	26	3.463 (2.307-5.196)	2.073 (.738-5.826)	.111
Negative	366	193	1	1	.167
**TB prophylaxis was given**					
Yes	97	11	1	1	0.000*
No	282	249	5.855 (3.2-10.7)	3.980 (1.875-8.448)	
**TB treatment recently**					
Yes	18	67	3.187 (2.410-4.216)	1.127 (.412-3.080)	.816
No	361	193	1	1	

Functional status during ART initiation was significant predictor of mortality. Patients in ambulatory functional status and bedridden are at increased hazard rate of death by 2.727 and 2.382 times than patients in working functional status respectively. This finding is consistent with many studies done in the past in Ethiopia. Study done in eastern Ethiopia show that risk of mortality was 4.09 time high for patients in bedridden functional status than working ones [22].

In this study, patients with fair and poor ART adherence are at high risk of death (2.169 and 1.887) times than those with good adherence. In line with this, the previous study by Bedru B. etal revealed that patients who have poor adherence were at risk of death by 3.92 than with those who have good adhered patients in Addis Ababa [19]. In relation to this, Gezahgn A. etal found that risk of death of poor adhered patients is 5.09 (95%CI, 5.51-49.48) than better adhered patients [26]. The possible explanation for high risk of death for patients with poor adherence should be studied further. Even though, adherence assessment technique is not as such reliable in our case (self reported), it was significant predictor of death. This could be an alarm for further study of the reason for adherence and also to increase the survivals of patients by establishing ways to good adherence. So, patients with poor ART adherence should be followed more frequently to decrease risk of death.

### Strength of the study

- Since lost, dropouts, transfer outs and transfer in were excluded so that mortality of the patient was not underestimated. This is true when patients with high risk of death among lost, dropouts and or transfer in or out.

### Limitations of the study

Using secondary data in which some important variables were not documented well and many opportunistic infections was presumed diagnosis.

## Conclusion

The Kaplan-Meier results showed that the general mean estimated survival time of patients after HAART initiation is improved.

Significant predictors of mortality after HAART initiation were: Lower baseline hemoglobin; Ambulatory and bed ridden functional status; Poor ART adherence; Advanced WHO clinical stage; Absence of recent TB prophylaxis; Unrecognized side effects; Persistent unexplained chronic diarrhea (>1 mon)

**Implications of the study findings** : Patients being on ambulatory and bedridden functional status should be assessed for other possible concomitant disease conditions and treated with closer follow up so as to minimize the risk of death. Patients with unexplained chronic diarrhea, oral candidacies and herpes zoster should be followed with special attention and these symptoms should be treated promptly. Important clinical characteristics of patients such as WHO staging, CD4 count, Hgb and other OIs should be documented correctly and regularly. The health team should access health status information of patients particularly on advanced age, jobless, being divorced, being in ambulatory and bedridden functional status, and other clinical results so that they follow these patients in especial consideration. Prospective study is necessary to get quality data by including variables which were not documented in routine healthcare practice like socio-economic status of patients, physician skill of treatment, BMI and other important laboratory investigations
